# Intimate partner violence: why does it matter for nurses?

**DOI:** 10.4069/whn.2025.08.26.1

**Published:** 2025-09-30

**Authors:** Sihyun Park

**Affiliations:** Red Cross College of Nursing, Chung-Ang University, Seoul, Korea

## Introduction

Intimate partner violence (IPV) is a serious global problem that threatens the health and safety of those affected. It is well documented that IPV leads not only to physical injuries [1] but also to a wide range of psychological and social consequences, including fear, depression, anxiety [2], suicidal ideation [3], social isolation [4], and, in the most severe cases, homicide [3].

The prevalence of IPV is alarmingly high. In the United States, for example, approximately 41% of women and 26% of men report experiencing some form of IPV during their lifetime [5]. Such high prevalence is not limited to the United States but is also observed worldwide. Studies have shown that 44.8% of women in Saudi Arabia [6], 66.0% of women in Turkey [7], 34.9% of women in South Korea [8], and 96% of women and 95.7% of men in Iran [9] have reported experiencing IPV. Although IPV affects all genders, being female and of younger age are significant risk factors [10]. Women are vulnerable regardless of employment status, with the likelihood increasing for those with children and those living in larger families [10].

Despite its higher prevalence compared to many other diseases or health conditions, and its devastating health and safety consequences, IPV has been relatively neglected within the discipline of nursing. This paper therefore reviews IPV in terms of its conceptual definitions and up-to-date intervention strategies, and discusses its implications for nursing.

## Definition of intimate partner violence and its subtypes

IPV refers to violent behaviors directed against intimate partners that cause physical, psychological, or sexual harm [11]. It can occur not only within current intimate relationships but also in former marital or dating partnerships. Conceptually, IPV is often categorized into three primary types: physical, psychological/emotional, and sexual violence. Physical violence refers to the intentional use of physical force against a partner, resulting in pain, injury, or disability. Such behaviors include hitting, slapping, punching, kicking, or burning, among others. Psychological violence, by contrast, refers to the intentional infliction of harm through verbal and nonverbal communication, such as threatening, insulting, intimidating, or humiliating. Sexual violence involves nonconsensual sexual acts or behaviors that are forced or attempted through coercion. This may include sexual harassment, threats of sexual coercion, or rape. Nevertheless, IPV is a far more complex phenomenon than can be captured by these three categories alone. As refining the concept allows for the development of more tailored and context-specific prevention and intervention strategies, researchers have increasingly sought to classify IPV into additional subtypes based on its patterns, tactics, and dynamics.

The Centers for Disease Control and Prevention [5] has included stalking as an additional type of IPV. Stalking is defined as “a pattern of behavior directed at a specific person that would cause a reasonable person to fear for the person’s safety or the safety of others; or suffer substantial emotional distress” [12]. Although stalking behaviors are not limited to intimate partner relationships and can occur in various interpersonal contexts, stalking by former intimate partners is recognized as the most prevalent and threatening form [13].

Beyond the more recognizable forms of IPV—such as physical, psychological, and sexual violence, as well as stalking—more subtle and less visible forms of violence also occur within intimate relationships. Because these forms often remain hidden, their scope and impact are less well understood, and in some cultural contexts, such behaviors against women are even justified. Thus, numerous efforts have been made to define and conceptualize such forms within the broader IPV phenomenon.

Johnson [14] distinguished two distinct types of IPV: “common couple violence” and “patriarchal terrorism” (more recently referred to as intimate terrorism), which reflect dynamics of gender power symmetry and asymmetry. The former, type common couple violence, arises from conflict within intimate relationships, where both partners may engage in physical or psychological aggression. Those who adopt this perspective often emphasize a gender-neutral view, suggesting that both perpetration and victimization can occur regardless of gender [15]. In contrast, patriarchal terrorism reflects a pattern of domination, typically characterized by men exerting power and control over women, rooted in broader social and cultural gender hierarchies [16]. Within this framework, coercive control has emerged as a critical element. Coercive control refers to the systematic restriction of a partner’s autonomy, freedom, and independence, thereby establishing dominance [16]. While coercive control may appear more subtle and less visible, it frequently co-occurs with physical, psychological, and sexual violence, and is often regarded as a precursor to more severe forms of IPV [17]. Feminist scholars have historically described IPV using terms such as “battered wives” or “violence against women” to highlight its gendered nature, situating male coercive control as a mechanism for maintaining dominance over women. From this perspective, such violence reflects men’s attempts to preserve authority and enforce traditional gender roles, commonly through emotional and financial control of their partners [18].

A newly recognized concept, social abuse, represents another subtle and largely invisible form of IPV. Social abuse refers to the intentional violation of an individual’s rights and needs for social belonging within an intimate relationship. It is enacted through four primary tactics: cutting off, interfering with, or surveilling the victim’s social relationships, and restricting their social engagement [4]. These tactics result in social isolation, which prevents victims from seeking help and reduces their likelihood of being identified and supported [4]. Recent technological developments, including social networking services, smartphones, and car-tracking applications, have further facilitated the enactment of social abuse. Through these digital tools, perpetrators can monitor victims’ movements, track their whereabouts, and surveil their daily lives more closely than ever before.

## Intervention strategies for intimate partner violence

### Existing intimate partner violence intervention programs and associated concerns

Numerous prevention and intervention programs have been developed for diverse populations and with varying objectives to address IPV. These programs can generally be categorized into four groups: (1) those targeting IPV victims (or potential victims) to reduce their risk of experiencing IPV, (2) those targeting IPV perpetrators (or potential perpetrators) to reduce the likelihood of perpetration, (3) those targeting couples already exposed to IPV to prevent further violence, and (4) those targeting bystanders to strengthen their capacity to intervene in IPV incidents. The majority of these programs have been developed in the United States and rely primarily on psychological intervention strategies [19].

Programs such as Telephone Support Services [20] and the My Plan Kenya app [21] aim to reduce IPV by supporting women at risk of, or currently experiencing, IPV. These interventions are designed to enhance women’s empowerment, improve health outcomes, increase help-seeking intentions and skills, strengthen the quality of romantic relationships, and improve safety preparedness, while simultaneously reducing psychological symptoms and experiences of victimization [19]. In contrast, programs such as the Sonke CHANGE (Community Health Action for Norms and Gender Equity) Intervention [22] and Substance Abuse-Domestic Violence treatment approach (SADV) [23] seek to prevent IPV by working with (potential) perpetrators. These interventions primarily target men in the general population as well as substance-dependent perpetrators. Their objectives include challenging perceptions of gender inequality and traditional norms, improving relationship quality, reducing risk behaviors, and preventing violence perpetration [19]. Additionally, programs such as GATHER [24] and the Domestic Violence Focused Couples Treatment Program [25] focus on couples already exposed to IPV, aiming to address relational dynamics and prevent further violence.

Despite their contributions, these programs raise several concerns. The first concern is victim-blaming. IPV prevention education that targets victims often implies that responsibility for violence lies with them, leading to blame when they are perceived as failing to respond effectively. Victim-blaming is a typical societal response to IPV across many cultural contexts, including South Korea. Victims who are blamed are less likely to seek external help and more likely to internalize guilt for their partner’s violence, which exacerbates psychological and emotional difficulties.

A second concern involves the gendered framework of many existing programs. These interventions frequently adopt a male-to-female violence model, providing perpetration-prevention education for men and victimization-prevention education for women. Such an approach reinforces gender-based assumptions about perpetrators and victims, neglects male IPV victims, and in some cases reduces men’s willingness to engage in IPV education.

A third concern arises with programs directed at couples already exposed to IPV. While these interventions can be effective in addressing relationship dynamics, they also carry risks for victims. Although safety may require victims to be physically separated from their abusers, such programs can provide perpetrators with additional opportunities to manipulate and control their partners under the guise of treatment or education. In light of these concerns, recent and innovative IPV interventions increasingly focus on bystanders, who can play a critical role in preventing and interrupting violence.

### A bystander intervention framework

Bystanders are individuals who witness or are aware of violence—such as family members, friends, or community members—and who have the potential to intervene in IPV situations. According to Latané and Darley’s model [26], bystanders typically progress through several steps when deciding whether to intervene: (1) becoming aware of the situation, (2) interpreting it as problematic, (3) assuming responsibility, (4) knowing how to act, and (5) taking action. However, it is often difficult for bystanders to move smoothly through these stages, as various barriers may impede the process. Such barriers include failure to notice due to limited knowledge, reluctance to assume responsibility, low perceived efficacy in intervening, audience inhibition, restrictive societal norms [27], and discouraging experiences from previous unsuccessful attempts to help [28]. To address these barriers, extensive bystander-focused programs have been developed to strengthen bystanders’ intentions to intervene, enhance intervention behaviors, and reduce obstacles to action. Bystander intervention programs originally emerged as part of broader efforts to combat campus crime, including sexual assault, following the passage of the Campus Sexual Violence Elimination (SaVE) Act in 2013 in the United States. The SaVE Act mandated prevention programs on the United States college campuses to raise awareness and prevent sexual assault. Consequently, most bystander intervention programs have been developed in the United States and primarily applied to college populations, with a particular emphasis on preventing sexual assaults [29].

## What nurses can do for intimate partner violence

Nurses are uniquely positioned to address and respond to issues of IPV. As healthcare professionals trained to care for an individual’s physical, mental, sexual, and social health, nurses are equipped not only to provide holistic care to victims of violence but also to serve as vital links between medical facilities and community resources. Because many IPV victims are isolated at home under the control of perpetrators or seek medical attention due to physical or sexual violence, nurses working in healthcare institutions or conducting home visiting programs are often among the first professionals able to recognize signs of IPV.

Unfortunately, IPV has long been relatively neglected within the nursing discipline, particularly in women’s health nursing, when compared to other illnesses and health concerns such as cancer care, pregnancy, and childbirth. Despite its devastating impact on women’s health and safety, along with its high prevalence and complex dynamics, IPV has often been treated as an uncomfortable subject, shaped by cultural, religious, and social norms. Women experiencing IPV have frequently been isolated or silenced under these circumstances, and in some cases even taught or encouraged to tolerate violence from their partners [18]. Nevertheless, nurses, who emphasize their closeness to patients and their dual roles of care and advocacy, have not always demonstrated effectiveness in recognizing, supporting, or understanding victims of IPV [30]. At times, the nursing profession has viewed violence against women as an uncomfortable yet inevitable cultural phenomenon, detached from health and nursing practice. It is now imperative, however, for nurses to actively address IPV as a critical health issue, assume responsibility for protecting victims, and play a proactive role in holding perpetrators accountable.

To guide effective and ethical intervention, I propose a structured five-step process. This model emphasizes both patient safety and the professional responsibilities of nurses as frontline responders. The first step is separation (removing victims from perpetrators). This involves safely separating the patient from the suspected perpetrator. Nurses should ensure private consultation opportunities where the patient can speak without fear of reprisal or surveillance. This protects confidentiality, reduces coercion, and allows victims to disclose sensitive information more freely.

The second step is assessment (evaluating IPV victimization). Once privacy is secured, nurses should conduct a systematic assessment of potential IPV. This includes screening for physical injuries, sexual assaults, psychological symptoms, and partner control. Employing IPV screening tools and sensitive communication strategies is essential to accurately understand the extent of victimization while maintaining a nonjudgmental stance. Since assessment data may serve as legal evidence, it should be carefully documented and securely stored.

The third step is confrontation (naming and informing). After recognizing signs of abuse, nurses have a duty to gently confront the issue by acknowledging the abusive nature of the relationship. This step does not involve judgment but instead validates patients’ experiences, affirming that what they are enduring constitutes IPV. Clear, empathetic communication can empower victims who may have normalized or minimized the violence.

The fourth step is planning (building safety strategies). In collaboration with the patient, nurses should develop individualized safety plans. These may include strategies for ensuring safety during emergencies, preserving evidence remaining on the body, preventing unwanted pregnancy, identifying support networks such as trusted family members and friends, and providing information about shelters and hotlines for victims and their children. Safety planning should be tailored to the victim’s circumstances, accounting for cultural, financial, and familial contexts. Respecting the victim’s choices is essential, as they are often the best judges of what will ensure safety for themselves and their children.

The fifth step is action (mobilizing support and intervention). This final step involves implementing appropriate measures based on the safety plan, in collaboration with the patient and in accordance with institutional protocols. Nurses should systematically collect medical evidence that may legally substantiate experiences of physical or sexual assault. They are also responsible for providing comprehensive nursing care to support both the physical and emotional recovery of the patient. In cases of sexual assault, this may include administering prescribed medications to prevent unwanted pregnancy. In addition, nurses can facilitate connections with police, social workers, advocacy organizations, and other law enforcement or community resources when appropriate. In this role, nurses act as advocates, bridging healthcare with community systems to promote both immediate safety and long-term recovery.

## Conclusion

This article has examined the complex issue of IPV and emphasized its critical implications for nursing practice, particularly in healthcare settings where nurses are often the first professionals to encounter victims. Nurses must be trained not only to provide holistic care for IPV victims but also to collect and document forensic evidence, support victims through psychological counseling, and collaborate effectively with legal and community resources. To achieve this, the integration of Forensic Nursing into undergraduate and graduate curricula is essential to equip nurses with the skills necessary for identifying IPV, safeguarding victims, and holding perpetrators accountable. Developing nursing diagnoses specific to violence and implementing evidence-based nursing care plans will further strengthen the profession’s capacity to intervene decisively in IPV cases. Through these efforts, nurses can play a pivotal role in preventing violence, protecting victims, and ultimately contributing to a safer and healthier community.
